# Self-assessed bowel toxicity after external beam radiotherapy for prostate cancer - predictive factors on irritative symptoms, incontinence and rectal bleeding

**DOI:** 10.1186/1748-717X-4-36

**Published:** 2009-09-21

**Authors:** Michael Pinkawa, Marc D Piroth, Karin Fischedick, Sandra Nussen, Jens Klotz, Richard Holy, Michael J Eble

**Affiliations:** 1 Department of Radiation Oncology, RWTH Aachen University, Pauwelsstrasse 30, 52072 Aachen, Germany

## Abstract

**Background:**

The aim of the study was to evaluate self-assessed bowel toxicity after radiotherapy (RT) for prostate cancer. In contrast to rectal bleeding, information concerning irritative symptoms (rectal urgency, pain) and incontinence after RT has not been adequately documented and reported in the past.

**Methods:**

Patients (n = 286) have been surveyed prospectively before (A), at the last day (70.2-72.0 Gy; B), a median time of two (C) and 16 months after RT (D) using a validated questionnaire (Expanded Prostate Cancer Index Composite). Bowel domain score changes were analyzed and patient-/dose-volume-related factors tested for a predictive value on three separate factors (subscales): irritative symptoms, incontinence and rectal bleeding.

**Results:**

Irritative symptoms were most strongly affected in the acute phase, but the scores of all subscales remained slightly lower at time D in comparison to baseline scores. Good correlations (correlation indices >0.4; p < 0.001 for all) were found between irritative and incontinence function/bother scores at times B-D, suggesting the presence of an urge incontinence for the majority of patients who reported uncontrolled leakage of stool. Planning target volume (PTV), haemorrhoids and stroke in past history were found to be independent predictive factors for rectal bleeding at time D. Chronic renal failure predisposed for lower irritative scores at time D. Paradoxically, patients with greater rectum volumes inside higher isodose levels presented with higher quality of life scores in the irritative and incontinence subscales.

**Conclusion:**

PTV and specific comorbidities are important predictive factors on adverse bowel quality of life changes after RT for prostate cancer. However, greater rectum volumes inside high isodose levels have not been found to be associated with lower quality of life scores.

## Background

External beam radiation is a well established curative treatment for prostate cancer. As demonstrated in randomized trials, higher doses may improve local control in a subset of these patients [1-3]. Gastrointestinal toxicity is known to be the dose-limiting toxicity [4]. Numerous reports already exist in the literature [5-16].

The standard for measures of toxicity consists of RTOG/EORTC or CTC morbidity scoring criteria, physician-based scoring systems that assign a toxicity grade based on symptoms reported by the patient [17-22]. Usually, a grading is reported without the actual symptoms [1,3,23-25]. In most cases, higher grades ≥ grade 2 indicate rectal bleeding. In a study by Goldner et al[8], 52 (in a total group of 166) patients were reported with grade 2-3 EORTC/RTOG late rectal side effects - the reason was rectal bleeding for 50 patients (96%). A recently published factor analysis of the EPIC (Expanded Prostate Cancer Index Composite) questionnaire revealed three separate factors within the bowel domain: bleeding, incontinence and irritative symptoms [26]. The information concerning irritative symptoms (rectal urgency, pain) and incontinence is not adequately documented and reported. A potential underestimation of symptoms results from lack of patient reporting. Quality of life questionnaires are used to compensate these problems. The EPIC was found to be more sensitive to changes in bowel toxicity through a course of radiotherapy and correlate well with RTOG/EORTC scores [27].

The aim of this study was to evaluate gastrointestinal quality of life changes after external beam radiotherapy for prostate cancer with discrimination of the factors concerning rectal bleeding, bowel incontinence and irritative symptoms. Patient-related and dose-volume-related factors were tested for a predictive value, respectively.

## Methods

This study was based on consecutive patients who were treated due to T1-3N0M0 prostatic carcinoma with three-dimensional conformal radiotherapy (3DCRT) in the years 2003-2006. The treatment was based on a treatment planning CT scan in supine position with a slice thickness of 5 mm. Patients were asked to have a full bladder for the planning computed tomography (CT) scan and each radiotherapy fraction. In all scans prostate volume, planning target volume (PTV), bladder and rectum were delineated by identifying the external contours. The rectum volume enclosed the region from the anal canal (including the anal canal) to the rectosigmoid flexure. The integral dose (AUC-area under the curve) was defined as the relation of the area under the dose-volume histogram curve to the total area, multiplied by 100. Treatment plans were calculated using a four-field box technique with 15 MeV photons and a multileaf collimator. The PTV was required to be enclosed by the 90% isodose relative to the ICRU reference point [28] with a margin of 1.5 cm in the anterior/lateral and 1 cm in the craniocaudal and dorsal directions to the CTV (prostate with or without seminal vesicles). The total dose to the prostate in the reference point was 70.2-72 Gy at 1.8-2.0 Gy daily fractions.

Patients have been surveyed prospectively before (time A), at the last day (B), two months (median, range 6 weeks-6 months) after (C) and sixteen months (median, range 12-20 months) after (D) radiotherapy using a validated questionnaire, the Expanded Prostate Cancer Index Composite (EPIC) [29]. Only patients with questionnaire results from both time A and time D have been included in the analysis, resulting in 286 (A), 200 (B), 252 (C) and 286 (D) questionnaires at the respective points in time. Nearly all patients who were addressed to respond before treatment agreed to take part in the evaluation (>98%). The response rate to the last questionnaire was 91%, so that 28 patients who responded before treatment were excluded from the analysis. Baseline patient characteristics are presented in Table 1.

**Table 1:** Baseline patient characteristics

patient age (years); median (range)	71 (45-84)
planning target volume (cm^3^); median (range)	336 (169-631)

PSA (ng/ml); median (range) ≤10 ng/ml; n (%)	8 (1.2-150) 178 (62)

biopsy Gleason score <7; n (%)	185 (65)

clinical T-stage ≤2a; n (%)	237 (83)

low risk (PSA≤10 ng/ml; Gleason score <7; clinical T-stage ≤2a); n (%)	106 (37)

intermediate risk (PSA 10-20 ng/ml or Gleason score = 7 or clinical T-stage 2b-c); n (%)	101 (35)

high risk (two risk factors or PSA>20 ng/ml or Gleason score >7 or clinical T-stage >2c); n (%)	79 (28)

neoadjuvant hormonal therapy; n (%)	90 (32)

any documented comorbidity;; n (%)	179 (63)

hypertension; n (%)	74 (26)

coronary heart disease; n (%)	76 (27)

diabetes; n (%)	34 (12)

chronic obstructive pulmonary disease; n (%)	31 (11)

haemorrhoids; n (%)	17 (6)

stroke in past history; n (%)	12 (4)

peripheral arterial disease; n (%)	10 (4)

chronic renal failure; n (%)	8 (3)

The bowel domain consists of 14 questions for function and the subjective assessment of the associated bother ("no problem" - "very small problem" - "little problem" - "moderate problem" - "big problem"). Questions were classified into irritative, incontinence and bleeding subscales. The multi-item scale scores were transformed lineary to a 0-100 scale, with higher scores representing better health-related quality of life.

The questionnaire was handed over to the patients personally by one of the physicians at time A, B and C. Number of questionnaires was the lowest at the end of radiotherapy (time B) because this point in time was limited to a single day (last radiotherapy fraction) and no second opportunity existed for a missed questionnaire. Patients presented in the department six to ten weeks after the end of treatment. Missed questionnaires in the acute phase (time C) and questionnaires one to two years after radiotherapy (time D) were sent to the patients with a return envelope. If a questionnaire was not returned within 4 weeks, patients were contacted by telephone and urged to complete it.

Statistical analysis was performed using the SPSS 14.0 (SPSS, Chicago, Ill), software. To explore statistical HRQOL score differences between different subgroups, the Mann-Whitney-U-test was used. The Wilcoxon's matched-pairs test was applied to determine longitudinal changes in specific subgroups of patients, the Friedman test to consider multiple comparisons. Contingency table analysis with the chi-square test was performed to compare treatment groups with respect to categorical variables. The Spearman correlation coefficient was used to test the correlation between two variables. In a forward stepwise logistic multivariate analysis, different risk factors were tested for their independence. Tested patient-related variables were the comorbidities (see Table 1) and a neoadjuvant hormonal therapy, dose-volume related variables were the PTV, rectum volume and the dose to the rectum. The dose to the rectum was described by the AUC and an additional significant dose level. Cut-off values were defined as parameters with the lowest p-value for differentiation between quality of life changes (volumes tested in 10 cm^3 ^intervals, relative doses in 5% intervals). All p-values reported are two-sided, p < 0.05 is considered significant.

## Results

Quality of life scores before radiotherapy and at different intervals after radiotherapy - with differentiation of irritative symptoms, incontinence and bleeding - are presented in Table 2. In the acute phase, irritative symptom subdomains were most strongly affected. Mean differences ≥10 points in comparison to baseline levels were also reported in incontinence subdomains. Though mean differences for bleeding remained <5 points at all intervals, they decreased statistically significantly. Depending on the symptom, 3-7% more patients reported their bowel symptoms to be a moderate/great problem in comparison to the portion before radiotherapy. While the largest differences (7%) concerned rectal urgency and increased frequency of bowel movements, the smallest differences (and the smallest percentage at time D) were found for bloody stools (Table 3).

**Table 2:** Mean quality of life scores (quartiles in brackets) and differences in relation to baseline scores

	**time A**	**mean diff**. **(A-B)**	**time B**	**mean diff**. **(A-C)**	**time C**	**mean diff**. **(A-D)**	**time D**
bowel function score	92 (89;96;100)	15	78 (68;82;93)	5	87 (79;93;96)	3	89 (86;93;100)

irritative function	89 (85;95;100)	18	72 (60;75;90)	6	85 (75;90;100)	3	87 (81;92;100)

incontinence function	97 (100;100;100)	10	88 (100;100;100)	4	93 (100;100;100)	4	93 (100;100;100)

rectal bleeding	99 (100;100;100)	4	95 (100;100;100)	2	97 (100;100;100)	3	96 (100;100;100)

bowel bother score	93 (93;100;100)	20	74 (54;82;96)	10	83 (71;93;100)	7	86 (79;96;100)

irritative bother	92 (88;100;100)	23	69 (44;75;94)	12	80 (69;94;100)	7	84 (75;94;100)

incontinence bother	97 (100;100;100)	13	86 (100;100;100)	10	88 (100;100;100)	6	91 (100;100;100)

rectal bleeding bother	99 (100;100;100)	4	95 (100;100;100)	3	96 (100;100;100)	3	95 (100;100;100)

p < 0.01 for all comparisons relative to baseline scores at time A (Wilcoxon's matched pairs test for individual comparisons and Friedman test for all comparisons in a line)

**Table 3:** Specific symptoms and associated bother

	**time A (%)**	**time B (%)**	**time C (%)**	**time D (%)**
rectal urgency ≥once a day	19	45	19*	14*

uncontrolled leakage of stool ≥once a day	3	7	4*	4*

loose or liquid stools ≥rarely	53	75	58*	60*

bloody stools ≥rarely	5	17	9	13

painful bowel movements ≥rarely	22	46	36	30

>two daily bowel movements	10	31	18	17

crampy pain in the abdomen or rectum ≥once a day	1	15	6	5

*moderate/big problem from:*				

rectal urgency	6	32	16	13

increased frequency of bowel movements	4	30	16	11

watery bowel movements	2	19	11	6

losing control of stools	1	12	8	5

bloody stools	0	3	2	3

abdominal/rectal pain	4	18	13	8

bowel habits overall	6	30	17	13

p < 0.01 for all comparisons relative to baseline scores at time A

*difference not significant (χ^2^-test) in relation to baseline percentage (time A)

Good correlations (Spearman correlation coefficients >0.4; p < 0.001 for all) were found between irritative and incontinence function/bother scores at times B-D, suggesting the presence of an urge incontinence for the majority of patients who reported to have symptoms of fecal incontinence (patients with vs. without rectal urgency ≥once a day reported uncontrolled leakage of stool ≥once a day in 20% vs. 1% at time D; p < 0.001). Correlation indices between irritative and rectal bleeding scores were found to be lower (Spearman correlation coefficients 0.25 and 0.30; p < 0.001 for all).

Dose-volume related and patient-related (comorbidities, neoadjuvant hormonal therapy) factors have a significant impact on quality of life score changes (Table 4). The dose-volume histogram reveals a paradoxic association with quality of life changes. Patients with a larger AUC-R, and specifically larger volumes within higher isodose levels (R70, relative rectum volume enclosed by the 70% isodose presented in Table 4; comparable results were found for cut-off values at 22%/25%/30% of the rectal volume within the 90%/80%/60%-isodose) reported smaller quality of life changes - irritative and incontinence subdomains were both found to be affected. However, in contrast to patients with a smaller PTV (mean difference: 0 points), patients with a larger PTV presented with lower rectal bleeding scores more than one year after treatment relative to baseline levels. Patients with a chronic renal failure were found to have significantly lower irritative scores in the chronic phase after RT. Haemorrhoids and stroke in past history were important predictors for rectal bleeding at time D.

**Table 4:** Impact of patient-related and dose-volume-related factors on quality of life score changes

	**factor**	**mean diff**. **(A-B)**	**mean diff**. **(A-C)**	**mean diff**. **(A-D)**
bowel function score	AUC-R≥50%	13 vs. 16	3 vs. 8	1 vs. 4†
	R70≥25%	15 vs. 16	4 vs. 12*	2 vs. 8*
	coronary heart disease	17 vs. 14	8 vs. 4†	3 vs. 3
	stroke in past history	10 vs. 15	3 vs. 5	7 vs. 3†
	chronic renal failure	30 vs. 15	15 vs. 5	11 vs. 3†

irritative function	AUC-R≥50%	16 vs. 20	4 vs. 8	1 vs. 5†
	R70≥25%	18 vs. 18	5 vs. 13†	2 vs. 9†
	chronic renal failure	41 vs. 18	8 vs. 6	12 vs. 3†

incontinence function	AUC-R≥50%	11 vs. 8	0 vs. 8†	2 vs. 6
	R70≥25%	10 vs. 8	3 vs. 12†	3 vs. 9†

rectal bleeding	PTV≥300 cm^3^	4 vs. 4	2 vs. 2	4 vs. 0*
	NHT	4 vs. 4	3 vs. 2	0 vs. 4*
	coronary heart disease	6 vs. 3	5 vs. 1†	4 vs. 2
	haemorrhoids	12 vs. 4	7 vs. 2†	9 vs. 3†
	stroke in past history	0 vs. 4	0 vs. 2	10 vs. 3*

bowel bother score	R70≥25%	19 vs. 27	8 vs. 22*	5 vs. 15*
	stroke in past history	9 vs. 20†	2 vs. 10	8 vs. 7
	chronic renal failure	37 vs. 19	11 vs. 10	21 vs. 6*

irritative bother	AUC-R≥50%	10 vs.15†	8 vs. 11	6 vs. 7
	R70≥25%	22 vs. 31	10 vs. 26*	6 vs.16
	chronic renal failure	48 vs. 23	15 vs. 12	22 vs. 7*

incontinence bother	R70≥25%	11 vs. 21	9 vs. 16	5 vs. 14†

rectal bleeding bother	PTV≥300 cm^3^	4 vs. 6	2 vs. 4	5 vs. 0†
	coronary heart disease	7 vs. 3	5 vs. 2*	3 vs. 4

only factors with significant impact on quality of life changes shown (actually significant differences marked with † p < 0.05 or *p < 0.01)

Haemorrhoids (and other parameters) have not been found to be predictive for rectal bleeding at time A. However, the factors haemorrhoids and NHT (neoadjuvant hormonal therapy) had an independent impact on greater irritative symptoms before RT in multivariate analysis (Table 5). Haemorrhoids clearly increased the probability to have a big/moderate problem with bowel habits overall. This increased risk remained detectable at all intervals after RT. As already presented in Table 4, actually greater quality of life *changes *after RT have not been found in the function/bother scores or the irritative function/bother subscores for patients with haemorrhoids.

**Table 5:** Impact of patient-related and dose-volume-related factors on symptoms/problems in multivariate analysis

	**factor**	**time A**	**time B**	**time C**	**time D**
		
		**HR [95%CI]/p-value/incidence with presence vs. absence of factor**			
painful bowel movements ≥rarely	R70≥25%	-	-	-	0.4 [0.2-0.9] p = 0.03 27% vs. 45%
	haemorrhoids	4.5 [1.6-12] p < 0.01 53% vs. 20%	6.3 [1.3-30] p = 0.02 77% vs. 43%	2.7 [1.0-7.4] p = 0.05 59% vs. 34%	-
	chronic renal failure	-	-	-	7.2 [1.4-36] p = 0.02 75% vs. 29%

bloody stools ≥rarely	PTV≥300 cm^3^	-	-	-	8.2 [2.4-28] p < 0.01 17% vs. 4%
	R70≥25%	-	-	-	0.2 [0.1-0.6] p < 0.01 11% vs. 23%
	haemorrhoids	-	-	3.5 [1.0-12] p < 0.05 24% vs. 8%	4.0 [1.2-13] p = 0.02 29% vs. 12%
	stroke in past history	-	-	-	7.2 [2.0-26] p < 0.01 42% vs. 12%

*moderate/big problem from:*					

bowel habits overall	R70≥25%	-	0.4 [0.2-0.9] p = 0.03 26% vs. 52%	0.4 [0.2-1.0] p < 0.05 15% vs. 32%	-
	haemorrhoids	8.1 [2.5-26] p < 0.01 29% vs. 5%	3.4 [1.0-11] p < 0.05 62% vs. 28%	5.9 [2.1-17] p < 0.01 53% vs. 15%	3.3 [1.1-10] p = 0.04 29% vs. 12%
	chronic renal failure	-	-	-	7.9 [1.9-33] p < 0.01 50% vs. 12%

rectal urgency	R70≥25%	-	-	0.3 [0.1-0.6] p < 0.01 13% vs. 35%	-
	NHT	4.3 [1.5-12] p < 0.01 11% vs. 3%	-	-	-
	haemorrhoids	5.2 [1.5-12] p = 0.03 18% vs. 5%	3.7 [1.2-12] p = 0.03 62% vs. 30%	-	3.4 [1.1-10] p = 0.03 29% vs. 12%
	chronic renal failure	-	-	-	4.9 [1.1-22] p = 0.04 28% vs. 12%

losing control of stools	age≥70 years	-	-	3.6 [1.0-12] p < 0.05 11% vs. 3%	-

bloody stools	haemorrhoids	-	-	-	7.0 [1.2-39] p = 0.03 12% vs. 2%

Without an influence already before RT, the multivariate analysis supports the independent influence of larger volumes within higher isodose levels (paradoxic effect) and chronic renal failure on late irritative symptoms; and a larger PTV, larger volumes within higher isodose levels, haemorrhoids and stroke in past history on rectal bleeding (due to an association of NHT with PTV, the effect of NHT has not been found to be independently predictive). The PTV was found to be significantly larger for patients with larger rectum volumes inside specific isodoses (mean 353 vs. 284 cm^3 ^with a R70 ≥25% vs. <25%, p < 0.001). Not a single patient (n = 80) with a PTV <300 cm^3^, without haemorrhoids or stroke in the past history reported to have rectal bleeding at time D. A significant effect of the patient age on bowel incontinence was only detectable at time C.

## Discussion

This study reports in detail bowel quality of life changes after 3DCRT, patient-related and dose-volume-related predisposing factors. In contrast to most other studies [1-3,11-13,25], patients were treated with a homogeneous dose and the same radiotherapy technique. Different dose levels usually coincide with changing treatment margins and radiotherapy techniques, implying possibly confounding variables. Patients after a radical prostatectomy, with a different anatomic situation, have not been included. Previous surgery can be predisposing for greater toxicity [30]. The factors irritation, incontinence and bleeding defined separate quality of life subscales. Usually reported RTOG/EORTC toxicity grades do not allow this perspective. Rectal urgency or incontinence is not considered in these studies [20].

Significant correlations have been found between acute and late quality of life changes, particularly considering the irritative and incontinence domains. As shown by Heemsbergen et al. [31] recently, consequential late damage (non-healing acute response progressing directly into a late effect) is a significant independent factor in the development of late gastrointestinal toxicity.

Rectal bleeding is usually the mostly feared toxicity and in focus of toxicity studies. It is considered a key dose-limiting end point in prostate radiotherapy [4]. In contrast to most other reports, this study does not evaluate a cumulative incidence of rectal bleeding since the end of treatment. The questionnaire asks about a specific period of four weeks. This study demonstrated that rectal bleeding is a subordinated problem in comparison to other problems from the patients' perspective. Especially in the acute phase (time B and C), irritative symptoms (urgency, frequency, pain) play the major role.

Incontinence was shown to be strongly associated with irritative symptoms. Nearly without exception, a daily incontinence was only reported with a simultaneously present rectal urgency at time D. Anorectal manometry pressures have not been documented in this patient population. Yeoh et al[32] reported significantly reduced anorectal manometry pressures 4 to 6 weeks after radiotherapy for patients with fecal incontinence. However, patients with incontinence were found to have normal basal and squeeze pressures one year after radiotherapy [33]. In a recently published study concerning anorectal function after radiotherapy for prostate cancer, a progressive deterioration of anorectal motor and sensory function has been shown. Rectal compliance progressively decreased and fecal sensitivity to distension increased after treatment [34].

These results focused on a median period of 16 months after treatment. Chronic effects several years after treatment might have additional effects on quality of life. In contrast to rectal bleeding, the cumulative incidence of fecal incontinence has been shown to increase even after more than five years [35]. The percentage of patients with fecal incontinence has been shown to be higher than the incidence of rectal bleeding requiring laser or transfusion in the Dutch dose-escalation trial in both treatment arms (68 Gy and 78 Gy total doses) [35], confirming the findings of our study.

Significant predisposing factors for both rectal bleeding subscale score changes and the symptom "rectal bleeding" in multivariate analysis were the PTV, haemorrhoids and stroke in the past history. A volume-effect is already very well known [9,10,36]. Patients with a stroke in the past history always take anticoagulative drugs - this is the most probable reason for the considerably increased tendency for bleeding. Other agents (clopidrogel/ticlopidine) or a higher aspirin dose is used in comparison with patients with coronary artery disease of peripheral artery disease. The association of anticoagulants with acute and late rectal bleeding has been shown in other studies before [37,38].

Haemorrhoids have been reported to be predictive for acute tenesmus and rectal bleeding after 3DCRT before [39]. Our study very well demonstrates the predisposition for irritative symptoms already before 3DCRT, so that quality of life *changes *did not differ significantly. However, the effect on rectal bleeding became only evident *after *3DCRT.

Higher volumes within higher isodose levels were associated with smaller quality of life changes in the irritative and incontinence subscales after 3DCRT. The multivariate analysis demonstrates the independence concerning irritative symptoms. This result may be associated with the specific dose constellation and treatment technique in our patient group. Yeoh et al[34] reported a significantly lower incidence of rectal urgency for patients after 2DCRT vs. 3DCRT in spite of a considerably larger PTV - these patients have likewise been treated with a four-field technique and doses not exceeding 72 Gy.

The reduction of irritative symptoms (urgency, frequency, pain) can be the consequence of a radiation effect on peripheral nerves. In the case of all nervous tissues, a dose of 60 Gy is associated with a less than 5% probability of injury, but this rises steeply with increased radiation dose. The pathogenesis involves progressive vascular degeneration, fibrosis and demyelination with loss of nerve fibres. The latency period ranges from 6 months to several years [40]. Comparably to an earlier development of erectile dysfunction [41], also usually considered as a chronic effect, differences have already been detected a few weeks after 3DCRT. Higher total doses, exceeding 72 Gy, can be expected to be associated with an increased risk of rectal ulcerations [35].

The association of larger rectum volumes within specific isodose levels with a paradoxically improved quality of life in the bowel domain was already found in a preliminary analysis of the first eighty patients [14]. This association was also found in an independent group of patients after permanent brachytherapy with I-125 as monotherapy for prostate cancer [42]. Quality of life scores have not been correlated with dose-volume histogram parameters for the rectum in the literature before. However, a validation of this association by other centres is needed.

Chronic renal failure is a rare comorbidity in a population of prostate cancer patients, with only 3% affected in our patient population. It has not been considered as a predisposing factor for toxicity before. This study has shown a dramatically increase risk of toxicity (specifically irritative symptoms), proven to be highly significant only more than one year after radiotherapy in spite of a low disease incidence. Metabolic effects must be postulated as the reason for this finding, but the actual mechanism is not clear (possibly slightly higher blood levels of toxic agents). This finding has to be taken into account in the treatment decision process for these patients.

## Conclusion

Irritative symptoms constitute the major problem for patients in the acute phase during and after external beam radiotherapy for prostate cancer. Irritative symptoms, incontinence and rectal bleeding are all affected in the chronic phase. Rectal bleeding - usually estimated to be the most worrying symptom - is bothering the patients less in comparison to irritative symptoms and incontinence. Depending on the symptom, 3-7% more patients reported their bowel symptoms to be a moderate/great problem in relation to baseline values before radiotherapy. Incontinence correlates significantly with irritative symptoms.

Planning target volume, haemorrhoids and stroke in past history were found to be independent predictive factors for chronic rectal bleeding. However, greater rectum volumes inside high isodose levels have not been found to be associated with lower quality of life scores.

## Competing interests

The authors declare that they have no competing interests.

## Authors' contributions

MP, MJE have made substantial contributions to conception and design; MP and KF have made substantial contributions to acquisition of data; MP, MDP, KF, SN, JK, RH, MJE to analysis and interpretation of data. MP has been involved in drafting the manuscript. MDP, KF, SN, JK, RH, MJE revised it critically for important intellectual content. All authors have given final approval of the version to be published.
